# Efficacy of direct adductor canal block in pain control and sparing opioid consumption after total knee arthroplasty: a randomized controlled trial

**DOI:** 10.1186/s12891-025-09087-9

**Published:** 2025-10-01

**Authors:** Jisu Park, Do Hyun Kim, Chong Bum Chang

**Affiliations:** 1 Department of Orthopaedic Surgery, Seoul National University College of Medicine, SMG- SNU Boramae Medical Center, Seoul, Republic of Korea; 2 Department of Orthopaedic Surgery, Seoul National University College of Medicine, Seoul, Republic of Korea; 3 Department of Orthopaedic Surgery, Seoul National University Bundang Hospital, 82, Gumi-ro 173 Beon-gil, Bundang-gu, Seongnam-si, Gyeonggi-do 13620 Republic of Korea

**Keywords:** Direct adductor canal block, Postoperative pain control, Opioid consumption, Total knee arthroplasty

## Abstract

**Background:**

The adductor canal block has been proven effective in controlling postoperative pain, but it requires additional space and manpower. In contrast, intraoperative or direct adductor canal block (D-ACB) is performed during the surgical procedure, eliminating additional time and cost. The purpose of this study was to evaluate the efficacy and safety of D-ACB. The authors hypothesized that adding D-ACB to periarticular injection (PAI) would help control postoperative pain and reduce opioid use after TKA without causing major side effects.

**Methods:**

Among patients scheduled to undergo primary TKA from September 2023 to February 2024, 38 patients in the PAI-alone group and 40 patients in the D-ACB + PAI group were eligible for analysis. The PAI-alone group received only PAI, while the D-ACB + PAI group received an additional ACB intraoperatively. Pain VAS scores and opioid consumption from the day of operation to postoperative day 5 were collected. Neurotoxicity and cardiotoxicity were considered major adverse events and were monitored along with postoperative motor weakness and fall-down events.

**Results:**

The D-ACB + PAI group used less opioid compared to the PAI-alone group. No major adverse effects were observed in either group during the perioperative period.

**Conclusion:**

Intraoperative direct ACB demonstrated an opioid-sparing effect when combined with PAI. By performing ACB intraoperatively, no additional space, cost, or manpower was required, and patients did not need an additional catheter for pain control. Direct ACB is a simple and safe procedure that can facilitate recovery after TKA.

**Trial registration:**

This study was retrospectively registered at the Clinical Research Information Service on April 5, 2024 (KCT0009311).

## Background

Pain control and rapid ambulation are important goals after total knee arthroplasty (TKA) [1, 2]. Various methods have been proposed to achieve these objectives. One option is adding perioperative peripheral nerve blockade [3–5]. Compared to traditional patient-controlled analgesia (PCA) using opioids, this regional anesthetic technique has shown higher patient satisfaction with fewer adverse events [5]. Although the femoral nerve block (FNB) has been used, it has been associated with impairment in quadriceps muscle power [6–9]. As an alternative, the adductor canal block (ACB) has gained attention for its effectiveness in controlling pain while sparing quadriceps muscle power [6, 7]. Conventional ACB is performed preoperatively or postoperatively by an anesthesiologist using ultrasound guidance, requiring additional space, time, cost, and an experienced specialist [10]. To overcome these drawbacks, intraoperative ACB has been introduced [11]. Intraoperative or direct ACB (D-ACB) is performed by the surgeon as part of the surgical procedure, eliminating the need for additional time and cost. However, literature on its efficacy is still insufficient. Another popular pain control option is the periarticular injection (PAI) of multimodal drugs. PAI effectively controls pain, reduces opioid consumption, and facilitates early ambulation during the perioperative period after TKA [12]. Several studies report the efficacy of combining conventional ACB with PAI, but no studies have compared concomitant D-ACB with PAI to PAI alone.

This study aimed to determine: (1) if adding D-ACB to PAI was effective in controlling pain and reducing opioid use after TKA; and (2) if D-ACB was safe to use. The authors hypothesized that the addition of D-ACB would help control postoperative pain and reduce opioid use in the early postoperative period after TKA. Additionally, we hypothesized that D-ACB could be used safely.

## Methods

This prospective, randomized controlled trial was conducted in accordance with the Declaration of Helsinki. It was approved by the institutional review board of our hospital (IRB No. B-2308-844-001) and registered at the Clinical Research Information Service (KCT0009311). Written informed consent was obtained from all patients for participation. This study was conducted in accordance with the Consolidated Standards of Report Trials guidelines [13].

### Study subjects

Among patients scheduled to undergo primary TKA by a single experienced surgeon at our hospital from September 2023 to February 2024, those with primary osteoarthritis aged 50 years or older were eligible for this study. The exclusion criteria were as follows: patients with inflammatory arthritis like rheumatoid arthritis; patients with traumatic or infectious arthritis; patients undergoing simultaneous bilateral TKA; patients with prior history of contralateral TKA within 3 months; patients undergoing revision TKA; patients who received general anesthesia during TKA. Eligible patients were divided into a periarticular injection-alone (PAI-alone) group and a direct adductor canal block with periarticular injection (D-ACB + PAI) group by block randomization during an outpatient visit 2–3 weeks before the operation date. A third party performed computer-generated block randomization at a ratio of 1:1.

**Fig. 1:**
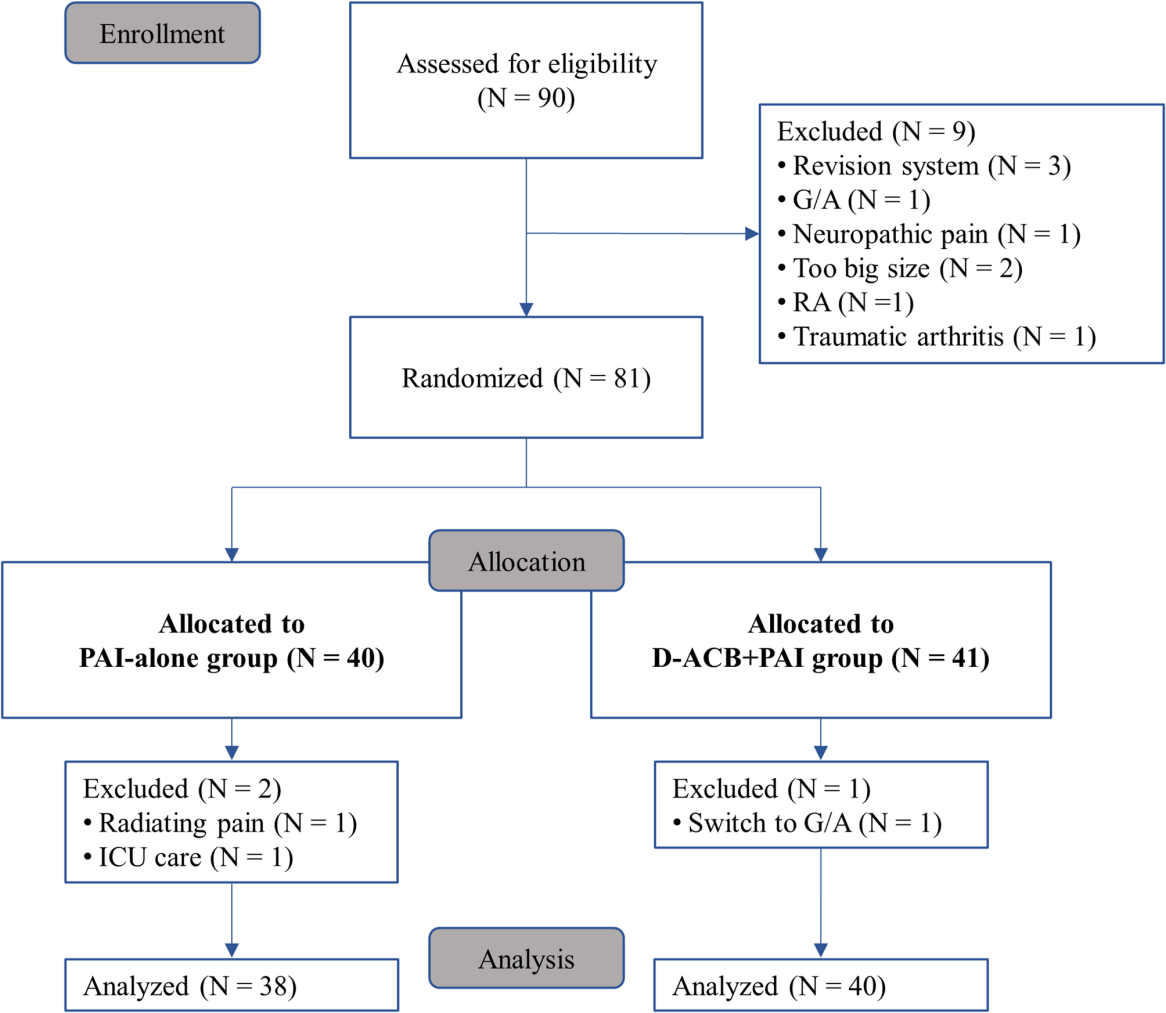
CONSORT flow diagram. G/A, general anesthesia; RA, rheumatoid arthritis; PAI, periarticular injection; D-ACB, direct adductor canal block; ICU, intensive care unit; N, number

Among 90 eligible patients, 9 were excluded after applying the exclusion criteria. 40 were classified into the PAI-alone group, and 41 were classified into the D-ACB + PAI group. After additionally excluding 2 patients from the PAI-alone group and 1 patient from the D-ACB + PAI group, 38 patients in the PAI-alone group and 40 patients in the D-ACB + PAI group were eligible for analysis (Fig. 1). The D-ACB + PAI group comprised more right-side TKA cases compared to the PAI-alone group. Except for the side of the surgery, there were no differences in demographics and preoperative characteristics between the two groups (Table 1).

**Table 1:** Demographic data

	PAI-alone (*N* = 38)	D-ACB + PAI (*N* = 40)	*P*-value
Sex (W: M, W %)	31:7 (81.6%)	34:6 (89.0%)	0.574
Age (mean ± SD, year)	72.8 ± 6.5	71.5 ± 6.3	0.376
Side (R: L, R %)	14:24 (36.8%)	27:13 (67.5%)	0.013
Height (mean ± SD, cm)	152.3 ± 7.4	153.3 ± 6.5	0.760
Weight (mean ± SD, kg)	64.3 ± 11.2	66.7 ± 10.6	0.340
BMI (mean ± SD, kg/m^2^)	25.8 ± 3.9	27.4 ± 3.5	0.179
FC (mean ± SD, degree)	8.9 ± 8.1	10.8 ± 7.9	0.285
FF (mean ± SD, degree)	118.9 ± 13.1	121.6 ± 12.6	0.359
Extensor power (mean ± SD, Nm)	35.9 ± 18.7	39.8 ± 14.3	0.159
Flexor power (mean ± SD, Nm)	26.1 ± 12.9	22.8 ± 11.7	0.435
KOOS (mean ± SD)	95.3 ± 23.1	101.5 ± 27.3	0.281
Equation 5D (mean ± SD)	9.9 ± 1.3	9.7 ± 1.6	0.346

*PAI* periarticular injection, *D-ACB* direct adductor canal block, *W* women, *M* men, *R* right, *L* left, *BMI* body mass index, *FC* flexion contracture, *FF* further flexion, *KOOS* knee injury and osteoarthritis outcome score, *EQ5D* EuroQol-5 dimension, *N* number, *SD* standard deviation

### Periarticular injection and adductor canal block

**Fig. 2:**
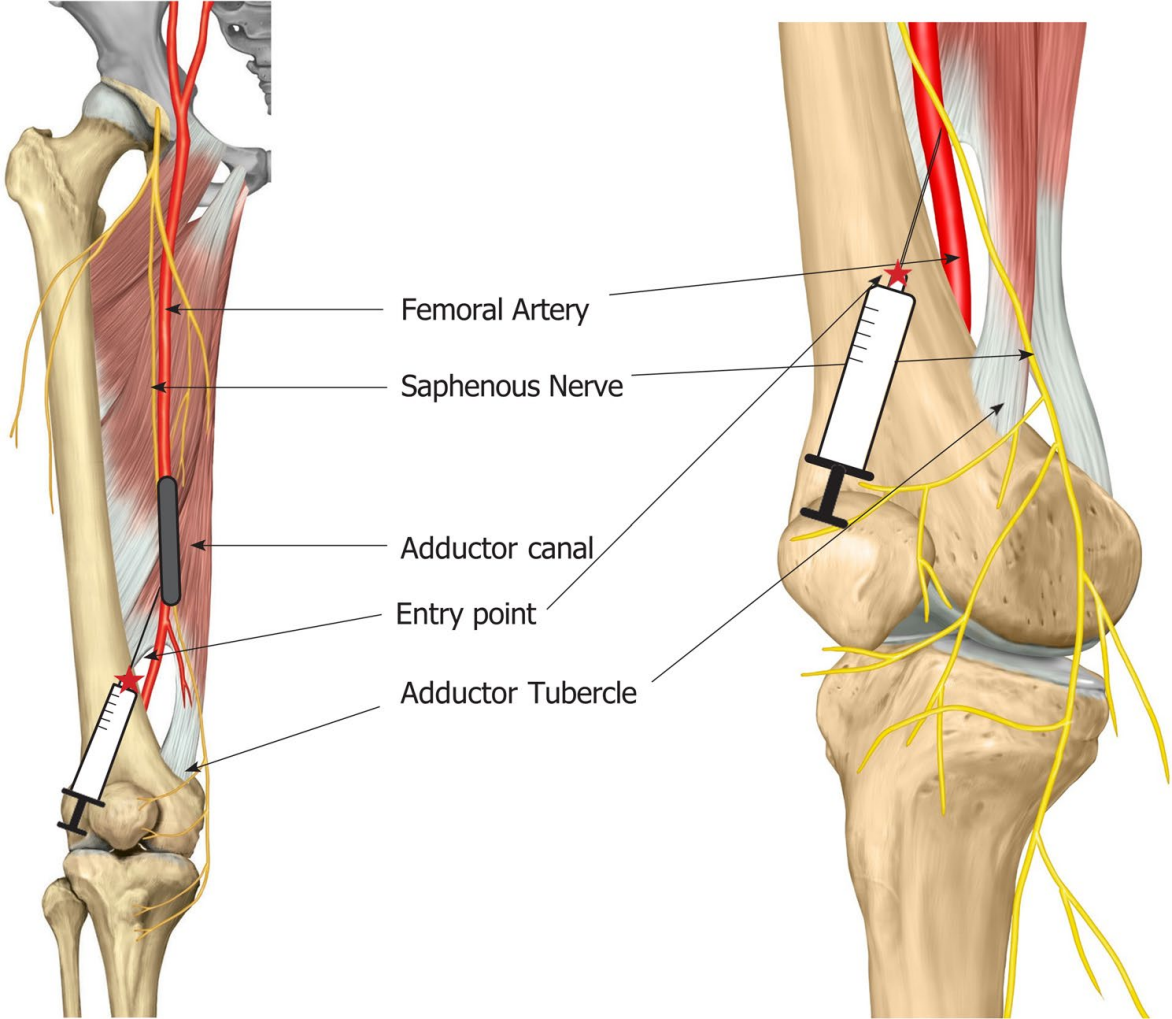
Diagram of D-ACB. The entry point (red star) of the needle is 8 cm above the joint line along the anteromedial border of the femur

**Fig. 3:**
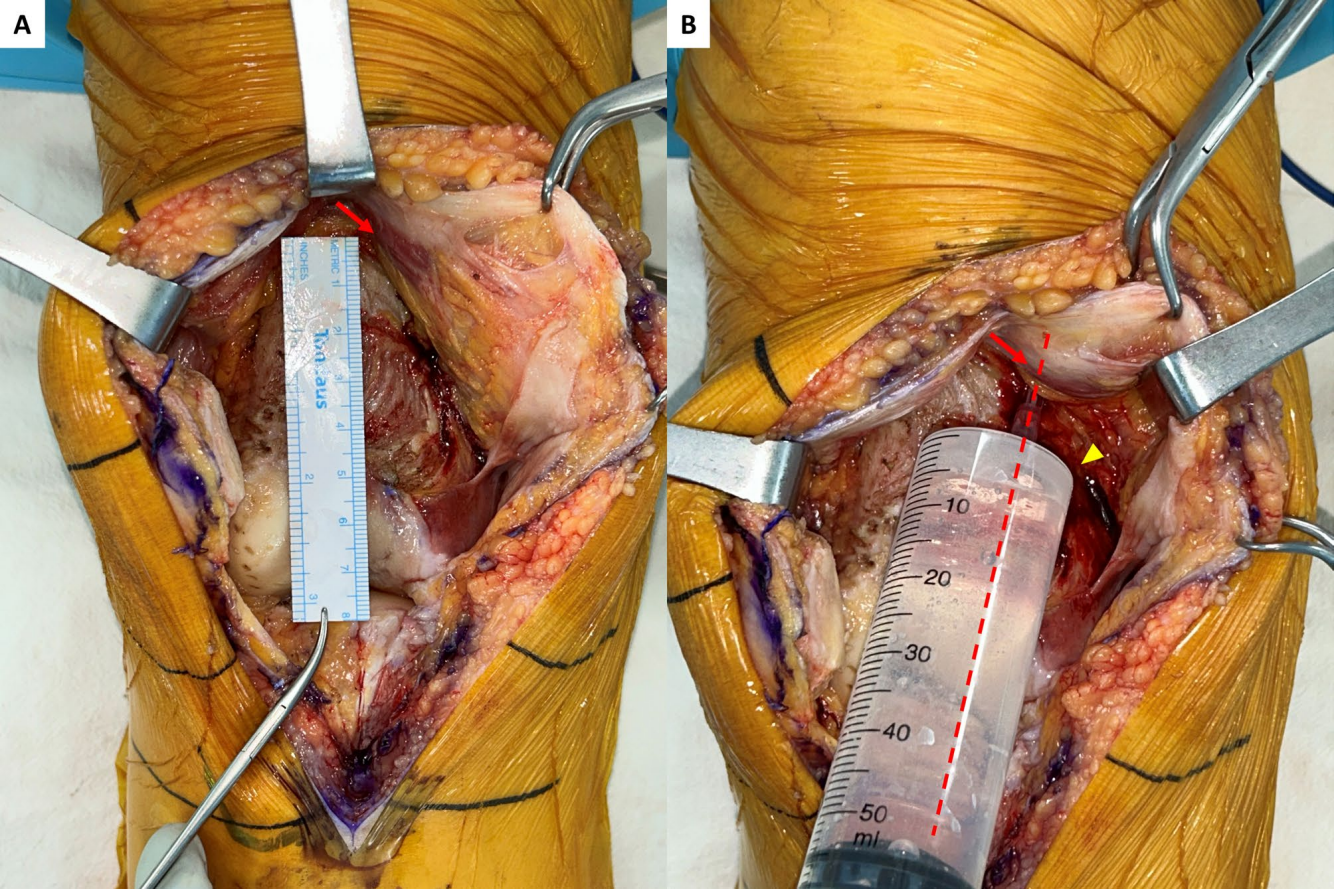
Intraoperative finding of D-ACB (coronal view). (**A**) The entry point (red arrow) of the needle is 8 cm above the joint line. (**B**) The needle is introduced at the entry point (red arrow). The needle is directed 6–10° medially. The yellow arrowhead represents the adductor tubercle

**Fig. 4:**
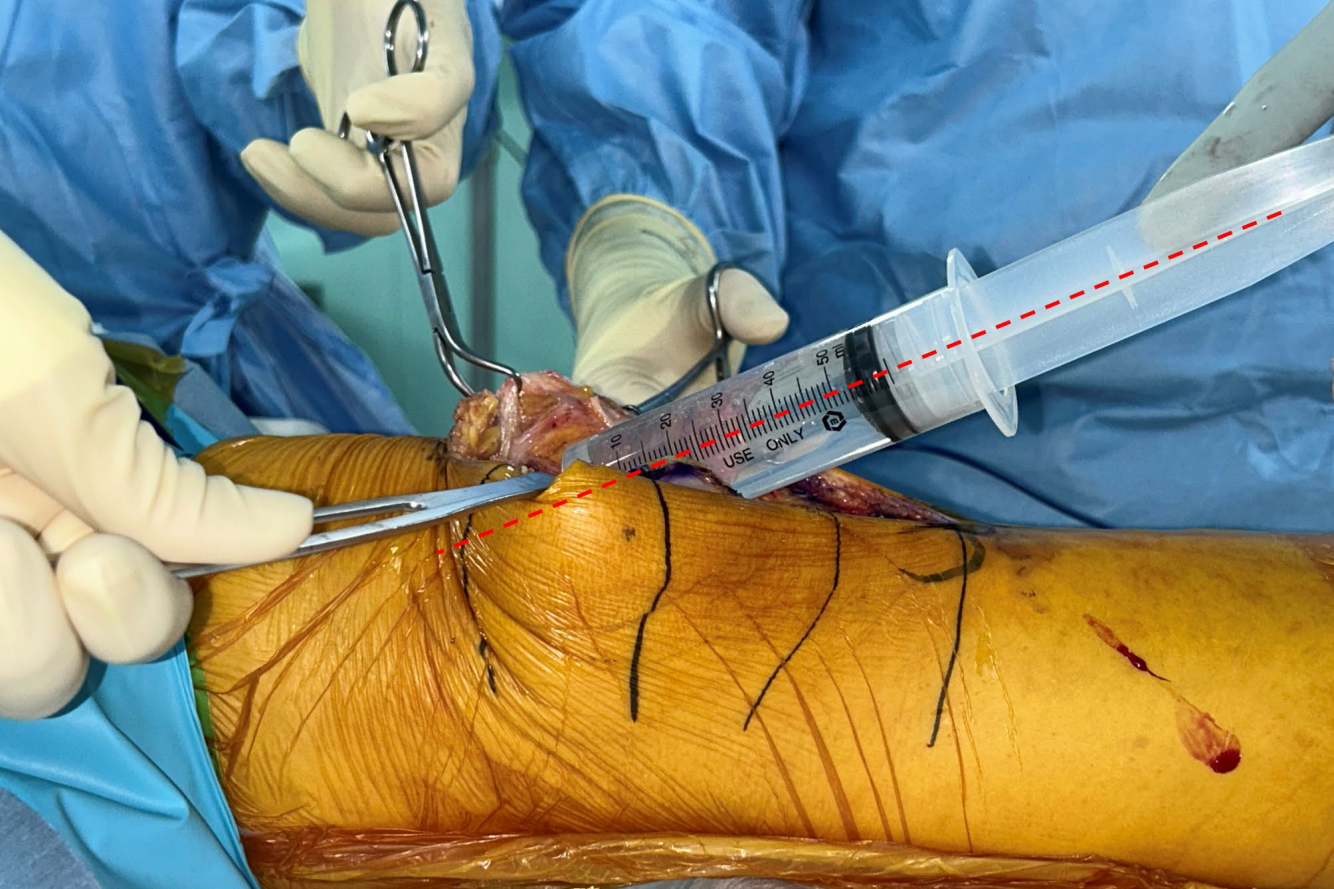
Intraoperative finding of D-ACB (sagittal view). The needle is directed 10–30° posteriorly

The cocktail for PAI and ACB was prepared with the following regimen: 300 µg of 1:1000 epinephrine, 10 mg of morphine sulfate, 30 mg of ketorolac, 300 mg of ropivacaine, 750 mg of cefuroxime, and 38.7 ml of normal saline, resulting in a total volume of 100 ml of the cocktail [14]. In the PAI-alone group, 50 ml of the cocktail was injected into the synovium, joint capsule, and peripatellar fat tissue. The remaining 50 ml was injected into the posterior capsule after cutting the distal femur and proximal tibia. In the D-ACB + PAI group, 25 ml of the cocktail was injected into the adductor canal for ACB, and the other 25 ml was injected into the synovium, joint capsule, and peripatellar fat tissue. For ACB, a 1.5-inch 18-gauge needle was used. The injection entry point was the medial gutter between the vastus medialis obliquus and medial femur, 8 cm above the distal end of the femur (Figs. 2, 3 and 4). The needle was angled 6–10°medially and 10–30°posteriorly, and advanced 6 to 7 cm [15, 16]. After aspiration to confirm that the needle was outside the vessel, 25 ml of the cocktail was injected. The remaining 50 ml was injected into the posterior capsule after cutting the distal femur and proximal tibia.

### Perioperative management and surgical technique

Except for the PAI and ACB procedures, the surgical technique and perioperative care protocol were identical between the two groups. The protocol followed was largely based on the Enhanced Recovery After Surgery (ERAS) protocol [17]. Clear fluids, including carbohydrate-containing drinks, were administered up to 2 h before surgery. Intravenous (IV) dexamethasone (10 mg) and multimodal oral analgesic drugs, consisting of 200 mg celecoxib, 650 mg acetaminophen in extended-release form, and 75 mg pregabalin, were given 1 h before the operation as preemptive medications. All patients received spinal anesthesia. Prior to inflating the tourniquet and after confirming the level of anesthesia, 1,000 mg of tranexamic acid (TXA) was administered intravenously. All surgeries were performed using a medial parapatellar approach, employing a modified gap technique, and aimed to achieve mechanical alignment. The prostheses used were posterior stabilized type implants with a fixed bearing system, and fixation was achieved using cement. Following closure of the joint capsule, an additional 1,500 mg of TXA was injected intra-articularly. Intra-articular or subcutaneous drainage was not utilized for any patients. All IV fluids were discontinued immediately upon commencing oral intake. Intravenous patient-controlled analgesia (PCA) was not employed in any patients. For pain management, multimodal oral analgesics, including 200 mg celecoxib, 650 mg acetaminophen ER, and 75 mg pregabalin, were used. Upon request, 100 mg of tramadol IV and 2.5 mg of morphine sulfate IV were administered as first- and second-line rescue analgesics, respectively. Postoperative IV dexamethasone (10 mg) was administered on postoperative day (POD) 1, followed by 5 mg on POD 2. To prevent deep vein thrombosis, an intermittent pneumatic compression device was applied during the perioperative period, and 100 mg of aspirin was administered for 3 weeks following TKA for chemical prophylaxis.

### Outcome assessment

Visual Analog Scale (VAS) scores for pain following TKA were assessed twice a day: at 8 AM and 8 PM. Opioid consumption from the day of the operation to POD 5 was recorded and converted into a morphine equivalent dose for analysis (100 mg of tramadol was converted into 20 mg of morphine using 0.2 conversion factor) [18]. Major adverse events included neurotoxicity and cardiotoxicity. Symptoms such as seizures, tinnitus, and blurred vision were monitored for potential neurotoxicity resulting from multimodal drug injection. For cardiotoxicity, symptoms such as chest pain, arrhythmia, and cardiac arrest were monitored. Additionally, postoperative motor weakness of the lower extremity and incidents of falling were observed.

### Statistical analysis

Sample size calculation was performed using G-power software (version 3.1). The sample size for an independent *t*-test was determined with a two-tailed effect size of 0.67, derived from the pilot study, an alpha error of 0.05, and a power of 0.8. The calculated sample size was 36 patients for each group. Considering an estimated dropout rate of approximately 10%, the study was designed to include 40 patients in each group, 80 patients in total.

All statistical analyses were carried out using R (version 4.2.2) and RStudio (version 2023.03.1 + 446). Descriptive statistical analysis was conducted, and data normality was assessed using the Shapiro-Wilk normality test. Homogeneity of variances was also verified. For categorical variables, either *Chi*-square or Fisher’s exact test was employed. Independent *t*-tests or Wilcoxon rank-sum tests were utilized for continuous variables. Statistical significance was defined as *P* <.05.

## Results

**Fig. 5:**
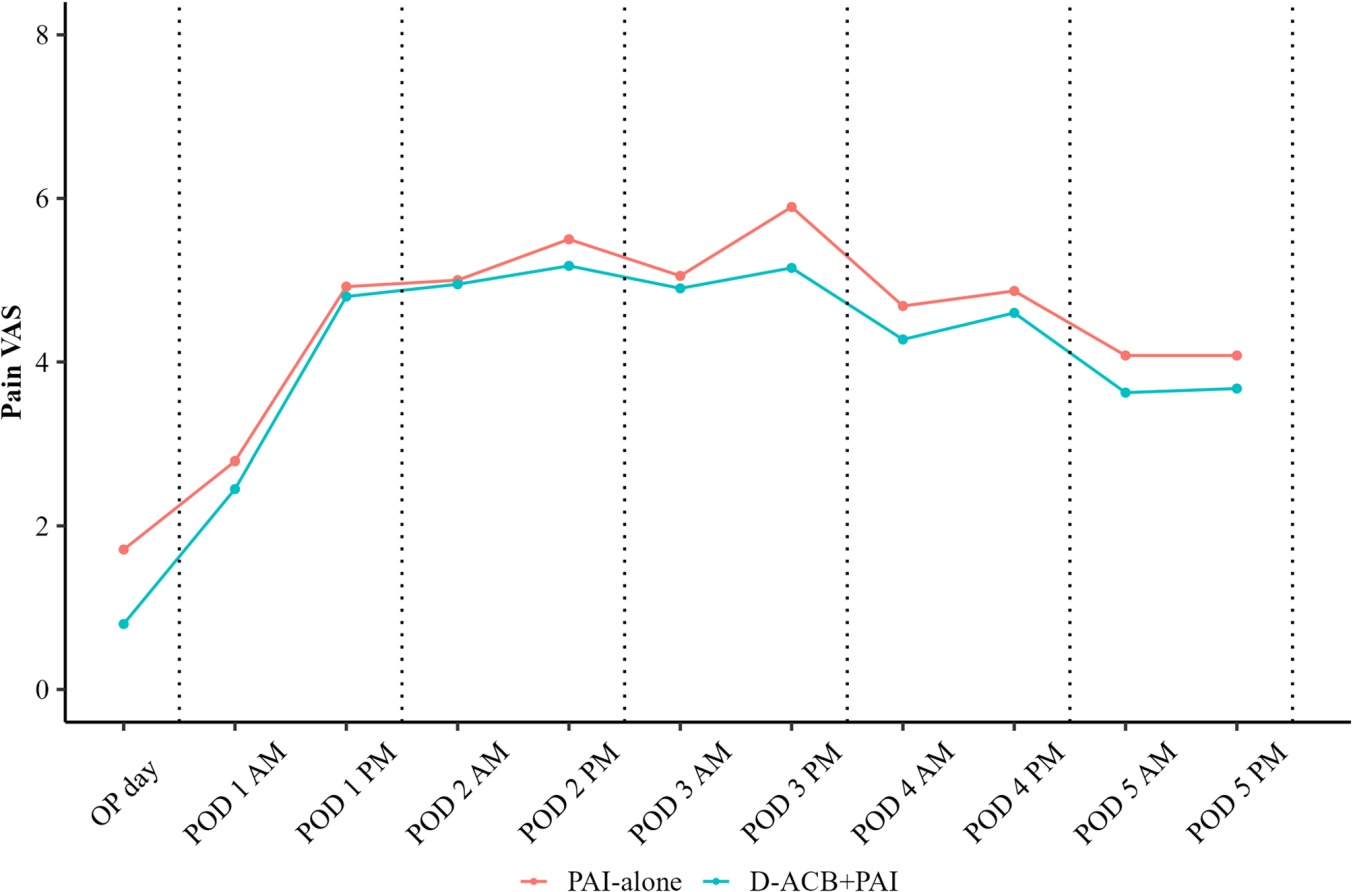
Pain VAS scores of perioperative periods. Pain VAS scores from the day of surgery to POD 5 were consistently lower in D-ACB + PAI group but showed no statistical significance. VAS, visual analogue scale; OP, operation; POD, postoperative day; PAI, periarticular injection; D-ACB, direct adductor canal block

**Fig. 6:**
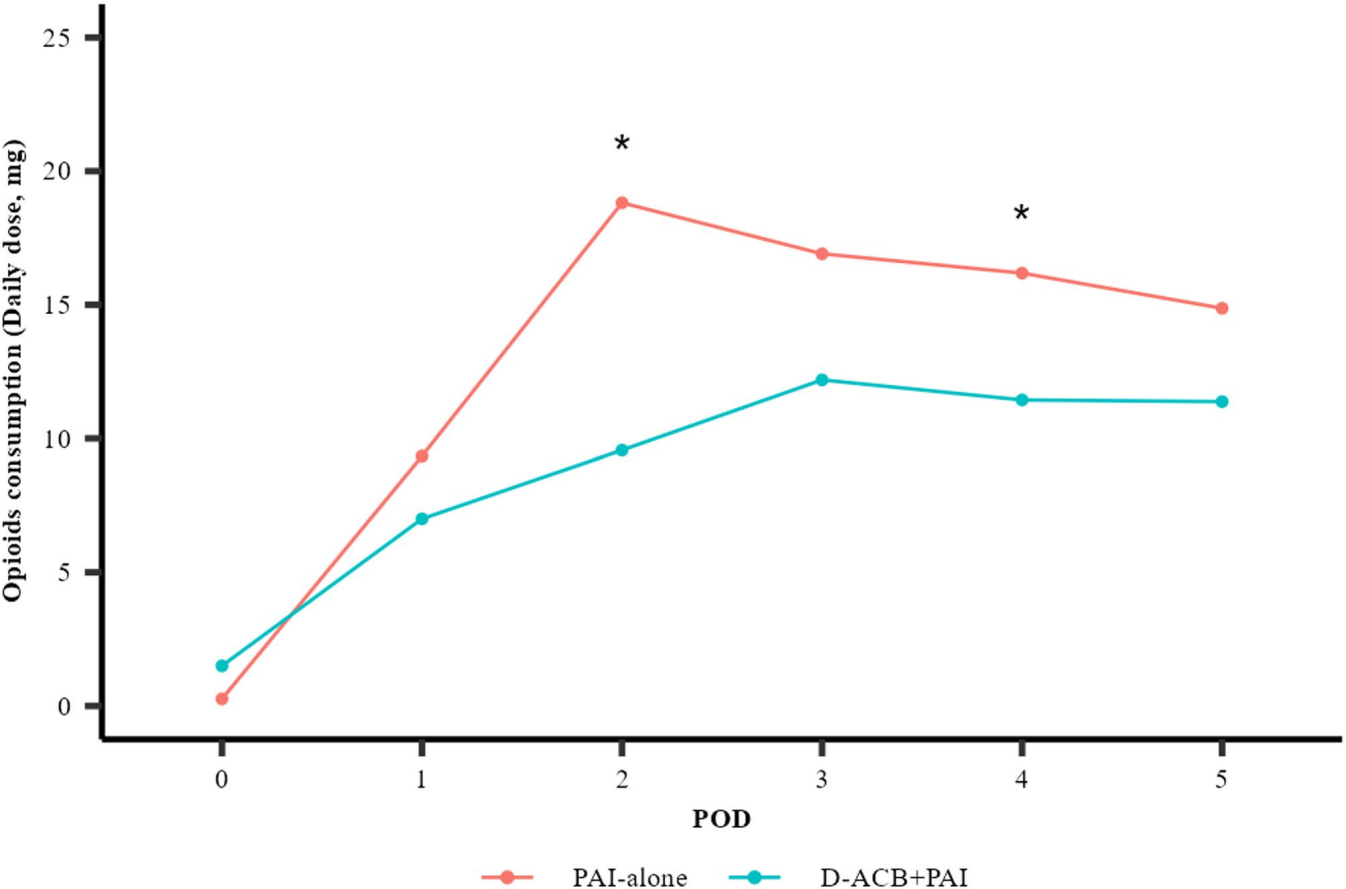
Daily opioid consumption. D-ACB + PAI group used significantly less opioid on POD 2 and 4 compared to PAI-alone group. D-ACB, direct adductor canal block; PAI, periarticular injection; POD, postoperative day. *P <.05

Pain VAS scores from the day of surgery to POD 5 were consistently lower in D-ACB + PAI group, although these differences did not reach statistical significance (Fig. 5). Opioid consumption on POD 2 and 4 was lower in the D-ACB + PAI group than in the PAI-alone group, with the greatest reduction observed on POD 2 (Fig. 6; Table 2).

**Table 2:** Comparison of daily dose of opioid

	Opioid daily dose (mean ± SD, mg)		
POD	PAI-alone	D-ACB + PAI	*P*-value
0	0.3 ± 1.6	1.5 ± 3.6	0.059
1	9.3 ± 10.7	7 ± 7.6	0.487
2	18.8 ± 13.8	9.6 ± 9.8	0.003
3	16.9 ± 11.6	12.2 ± 10.4	0.071
4	16.2 ± 9.7	11.4 ± 9.7	0.032
5	14.9 ± 11.7	11.4 ± 11.4	0.176

*PAI* periarticular injection, *D-ACB* direct adductor canal block, *POD* postoperative day, *SD* standard deviation

No major adverse effects were observed in either group during the perioperative period. None of the patients in either group reported lower extremity motor weakness post-surgery, and no incidents of falling were recorded.

## Discussion

Direct ACB is performed by surgeon during operative procedure and involves injection more distally than conventional ultrasound-guided adductor canal block (US-ACB). Several cadaveric and imaging studies, such as CT or MRI-based investigations, have demonstrated the feasibility of intraoperative ACB [11, 16, 19]. It is a simple and less time-consuming technique, although there is limited research on its efficacy so far. According to published retrospective studies, D-ACB has shown superiority over US-ACB in terms of pain VAS score shortly after surgery [15, 20, 21]. Greenky et al. conducted a randomized controlled trial (RCT) comparing D-ACB to US-ACB, and reported that D-ACB was not inferior to US-ACB regarding patient satisfaction, opioid consumption, and patient-reported outcomes [10]. While several studies have compared D-ACB to US-ACB, to our knowledge, our study represents the first RCT utilizing D-ACB and evaluating its efficacy when combined with PAI compared to PAI-alone. The primary findings of our study were: (1) the D-ACB + PAI group used less opioids but reported similar levels of pain VAS score compared to the PAI-alone group; and (2) D-ACB could be safely employed.

By adding intraoperative adductor canal block to periarticular injection, comparable pain control was achieved with reduced opioid usage. While previous studies have not directly compared D-ACB + PAI to PAI-alone, several have examined US-ACB + PAI versus PAI-alone. A meta-analysis by Ma et al. found that US-ACB + PAI led to improved ambulation compared to PAI-alone but did not show advantages in pain control or opioid consumption [22]. In a more recent meta-analysis by Fillingham et al., US-ACB + PAI was superior to PAI-alone in terms of pain VAS scores during the first 24 h after TKA, yet there was no difference in opioid consumption [23]. Additionally, Nader et al. conducted an RCT demonstrating the pain-relieving and opioid-sparing effects of combining US-ACB with PAI [24]. In our study, we observed an opioid-sparing effect of D-ACB. Considering the current emphasis on reducing opioid use following TKA, it is encouraging that such reductions can be achieved with the addition of a simple procedure. D-ACB offers additional advantages as a time- and cost-effective method, as it does not necessitate extra space or personnel [15]. There is a theoretical advantage of continuous catheter ACB for prolonged pain control over single-shot ACB. However, a recent meta-analysis showed that continuous ACB offered no benefit for postoperative pain levels, analgesic use, or functional recovery [25]. Instead, continuous ACB was found to be associated with a higher rate of block-related complications. Furthermore, the absence of a need for an additional catheter in D-ACB promotes greater mobility during the perioperative period, aligning with the principles of the ERAS society [17].

An intriguing finding in our study was that the disparity in opioid consumption emerged on POD 2 rather than on the operative day or POD 1. This outcome contradicted our initial hypothesis, which anticipated differences in the early postoperative periods. This result may be attributed to the multimodal drug cocktail used for ACB in our study. Previous studies have typically employed a single drug (such as bupivacaine) for the injection [26–30]. Given that ropivacaine has similar duration of effect with bupivacaine, the observed opioid-sparing effect on POD 2 appears to stem from the inclusion of multimodal drug agents in the injection [31, 32]. Similarly, Stith et al. compared multimodal agent regional anesthesia to single-agent regional anesthesia (bupivacaine only) and demonstrated the prolonged analgesic effect of multimodal agent blocks [33]. Thus, it seems feasible to mitigate rebound pain after PAI by supplementing D-ACB with multimodal agents.

No major adverse effects were observed in this study. Given that ACB was performed without ultrasound guidance, safety was a primary concern. Injection of agents like bupivacaine and ropivacaine directly into vessels can pose risks of neurotoxicity and cardiotoxicity. However, by aspirating the syringe before injection, these potential adverse events could be mitigated. A cadaveric study by Pepper et al. found that D-ACB was accurate and safe, with no vascular penetration [11]. Another concern was the potential blockade of motor branches to the quadriceps muscles, leading to decreased motor power and possibly fall-down in the perioperative period. Since ACB is performed at a more distal point than femoral nerve block (FNB), it may be possible to avoid motor nerve blockade, which typically occurs at the proximal part of the femur. As a result, less quadriceps weakness has been reported with ACB compared to FNB [6–9]. However, it’s important to note that not all motor nerves to the quadriceps branch out at the same level. According to a cadaveric study by Page et al., the motor nerve to the vastus medialis branches most distally, at the mid-femoral level [34]. Since this is typically the point of ultrasound-guided ACB, there remains a possibility of motor blockade. Adoni et al. compared conventional ACB with more distal ACB and found less motor blockade of the vastus medialis with the distal approach [35]. In D-ACB, the injection is performed even more distally than in conventional ACB, reducing concerns about motor weakness. The absence of postoperative motor weakness complaints in our study suggests the motor power-sparing effect of D-ACB.

There are several limitations to this study. First, since the injections were performed without ultrasound guidance, there is a possibility that the multimodal agents were not injected properly into the adductor canal. However, previous cadaveric studies have demonstrated the feasibility of D-ACB. Additionally, despite the lack of imaging confirmation that the needle was in the adductor canal, we believe that our results sufficiently demonstrate the efficacy of D-ACB, given the significant reduction in opioid use with no adverse events. Second, long-term outcomes were not measured in this study. However, since regional anesthetic techniques primarily affect short-term outcomes, perioperative observation may be sufficient to assess the value of D-ACB. Third, this study did not address functional outcomes. Ambulation, muscle power, and patient satisfaction were not primary outcomes of our study. Since our study primarily focused on pain and opioid consumption, these functional outcomes were not the main focus. Further evaluation of the functional aspect would be warranted.

## Conclusion

The addition of intraoperative D-ACB to PAI allowed for comparable pain control with reduced opioid usage. Moreover, no adverse events were observed during the perioperative period. Performing ACB intraoperatively eliminated the need for additional space, cost, and manpower, as well as the requirement for an additional catheter for pain management. Direct ACB emerges as a simple and safe procedure that can enhance recovery following TKA.

## Data Availability

The datasets used and/or analyzed during the current study are available from the corresponding author on reasonable request.

## Abbreviations

TKA: Total knee arthroplasty
PCA: Patient-controlled analgesia
FNB: Femoral nerve block
ACB: Adductor canal block
D-ACB: Direct adductor canal block
US-ACB: Ultrasound-guided adductor canal block
PAI: Periarticular injection
IV: Intravenous
TXA: Tranexamic acid
POD: Postoperative day
VAS: Visual analog scale
RCT: Randomized controlled trial
